# Fear memory formation can affect a different memory: fear conditioning affects the extinction, but not retrieval, of conditioned taste aversion (CTA) memory

**DOI:** 10.3389/fnbeh.2014.00324

**Published:** 2014-09-30

**Authors:** Gil Joels, Raphael Lamprecht

**Affiliations:** ^1^Sagol Department of Neurobiology, Faculty of Natural Sciences, University of HaifaHaifa, Israel; ^2^Department of Biology, Faculty of Natural Sciences, University of HaifaHaifa, Israel; ^3^Center for Gene Manipulation in the Brain, University of HaifaHaifa, Israel; ^4^Center for Brain and Behavior, University of HaifaHaifa, Israel

**Keywords:** learning and memory, memory extinction, memory retrieval, cued fear conditioning, conditioned taste aversion

## Abstract

The formation of fear memory to a specific stimulus leads to subsequent fearful response to that stimulus. However, it is not apparent whether the formation of fear memory can affect other memories. We study whether specific fearful experience leading to fear memory affects different memories formation and extinction. We revealed that cued fear conditioning, but not unpaired or naïve training, inhibited the extinction of conditioned taste aversion (CTA) memory that was formed after fear conditioning training in rats. Fear conditioning had no effect on retrieval of CTA memory but specifically impaired its extinction. Extinguished fear memory, after fear extinction training, had no effect on future CTA memory extinction. Fear conditioning had no effect on CTA memory extinction if CTA memory was formed before fear conditioning. Conditioned taste aversion had no effect on fear conditioning memory extinction. We conclude that active cued fear conditioning memory can affect specifically the extinction, but not the formation, of future different memory.

## Introduction

Ample studies have shown that when a specific sensory stimulus occurs together with a fearful event, long-term fear memory to the stimulus is formed (LeDoux, 2000). The formation of fear memory to a specific stimulus is useful for the subject in order to avoid subsequent encounter with it and to adjust the behavior. In this study we were interested to explore whether fear learning might affect the formation and extinction of different memories. Alteration of different memory formation and extinction after fear learning may serve to adjust memory performance and behavior in the changing fearful environment.

Memory extinction is one mechanism whereby the behavioral response is kept proportional to the situation. Extinction of memory occurs when the conditioned stimulus (CS) cues are presented alone without the unconditioned stimulus (US; Pavlov, 1927). It does not reflect forgetting of the original learning (e.g., the fearful event), but rather relearning of a new association of the CS with the absence of the original reinforcement (Rescorla, 1996). An optimal rate of extinction is required for the normal function and survival of the organism. Facilitated memory extinction may lead to an incorrect attribution of the stimulus and may lead to dangerous situations that may result in death or injury. Impairment of extinction is associated with behavioral dysfunction and with brain disorders such as in post-traumatic stress disorder (Milad et al., 2008, 2009; Norrholm et al., 2011; Milad and Quirk, 2012).

Toward exploring the possibility that fear memory may affect the formation and extinction of different memory we study the effects of cued fear conditioning on the formation and extinction of conditioned taste aversion (CTA) memory. In cued fear conditioning an animal forms fear memory to a neutral stimulus (in the current study tone- CS) paired with a fearful event (footshock- US) (Fanselow and LeDoux, 1999; LeDoux, 2000; Davis and Whalen, 2001; Maren, 2001, 2005; Schafe et al., 2001; Sah et al., 2003; Rodrigues et al., 2004; Johansen et al., 2011). In CTA an organism learns to avoid a taste (CS) if the first encounter with that taste is followed by malaise (US) (Garcia et al., 1955; Bures et al., 1998; Rosenblum, 2008).

Fear memory formation may induce changes in neurons within specific brain regions that affect the formation and extinction of future different memories. Cued fear conditioning and CTA memories formation and extinction are subserved by overlapping brain regions and therefore may be suitable behavioral paradigms for studying relationships between different memories. Fear conditioning leads to changes in responses to the CS in the lateral and central nuclei of the amygdala (LA and CE), auditory thalamus and auditory cortex (e.g., Gabriel et al., 1975; Edeline and Weinberger, 1992; Lennartz and Weinberger, 1992; Quirk et al., 1995, 1997; Ciocchi et al., 2010; Haubensak et al., 2010). These areas are needed for fear memory formation as lesions and functional ablations of these regions and inhibitions of molecular activity in these areas impair fear conditioning memory (e.g., Muller et al., 1997; Rodrigues et al., 2004; Ciocchi et al., 2010; Johansen et al., 2011). Evidence show that extinction of fear memory is mediated by the prefrontal cortex as prefrontal cortex lesions and molecular activity inhibition in prefrontal cortex lead to a selective deficit in extinction (Sotres-Bayon and Quirk, 2010; Maroun, 2013). Moreover, neuronal activity in infralimbic subregion (IL) of the medial prefrontal cortex (mPFC) changes with extinction (Milad and Quirk, 2002). The IL-mPFC may mediate fear memory extinction by controlling downstream areas such as the lateral subdivision of central amygdala nucleus and the intercalated cell masses (ITCs; McDonald et al., 1996; Likhtik et al., 2008). In addition, extinction involves depotentiation of excitatory pathways in LA (Kim et al., 2007) and extinction of fear-potentiated startle is blocked by infusion of an NMDA antagonist into the amygdala (Falls et al., 1992). Acquisition of CTA changes the pattern of activity in response to the taste CS in the nucleus of the solitary tract (NTS), parabrachial nucleus (PbN), amygdala and insular cortex (IC) (e.g., Chang and Scott, 1984; Houpt et al., 1994; Swank and Bernstein, 1994; Welzl et al., 2001; Yamamoto, 2006; Moran and Katz, 2014). Permanently or transiently inactivating the PbN impaired the acquisition of a CTA (e.g., Spector et al., 1992; Agüero et al., 1993; Bielavska and Bures, 1994; Grigson et al., 1997) and damaging the amygdala produced impairments in CTA but inconsistently depending on the paradigm and type of lesions (e.g., Nachman and Ashe, 1974; Lasiter and Glanzman, 1985; Simbayi et al., 1986; Yamamoto et al., 1995; Schafe and Bernstein, 1996; Morris et al., 1999; Lamprecht and Dudai, 2000; Welzl et al., 2001). Lesions of the thalamus including the parvicellular part of the ventroposterior medial nucleus (VPMpc) can attenuate the acquisition of CTA (Loullis et al., 1978; Lasiter, 1985; Lasiter et al., 1985; Yamamoto et al., 1995). Conditioned taste aversion and taste memory are influenced by damaging the IC and the IC is critically involved in taste memory consolidation, maintenance and retention (e.g., Braun et al., 1972; Dunn and Everitt, 1988; Bermudez-Rattoni and McGaugh, 1991; Gallo et al., 1992; Rosenblum et al., 1993). The IL-mPFC and the basolateral amygdala (BLA) nucleus have been shown to play a role in extinction of CTA memories (e.g., Bahar et al., 2003; Akirav, 2007).

The aforementioned observations show that mutual brain regions subserve both fear conditioning and CTA memory formation (e.g., amygdala) and extinction (e.g., prefrontal cortex and amygdala). We therefore investigate the possibility that fear memory formation affects other memories by studying the effects of fear conditioning on CTA memory formation and extinction.

## Materials and methods

### Animals

Male Sprague Dawley rats (250–300 g), were used in the study (Harlan Laboratories). Rats were housed separately at 22 ± 2°C in a 12 h light/dark cycle. Water and food were available *ad libitum* unless otherwise indicated. Behavioral experiments were approved by the University of Haifa Institutional Committee for animal experiments in accordance with National Institutes of Health guidelines.

### Fear conditioning

**Figure 1:**
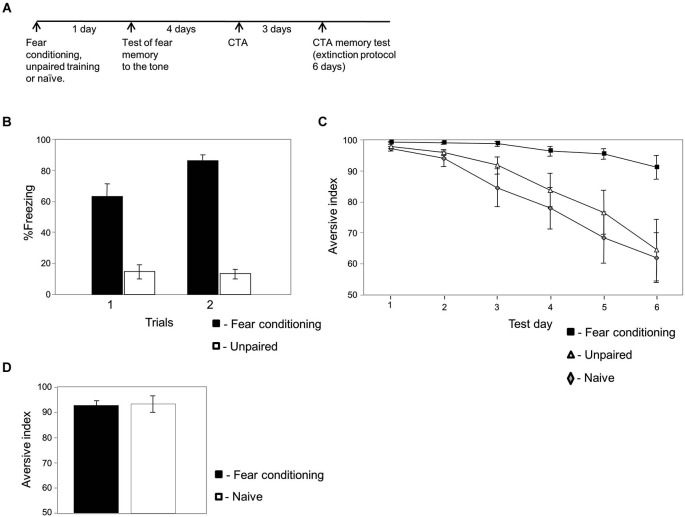
**Fear conditioning affects conditioned taste aversion memory (CTA) extinction**. **(A)** Experimental timeline: animals were trained for fear conditioning (*n* = 15), unpaired (*n* = 13) or left naïve (*n* = 20). The next day they were tested for fear memory to the tone. Four days later the animals were subjected to CTA training. Three days later they were tested for taste memory once each day for 6 days. **(B)** Freezing during fear memory test is significantly higher in fear conditioned animals compared to unpaired trained animals (χ^2^_(1)_ = 87.779; *p* < 0.003). This result shows that fear memory to the tone was formed in the fear conditioned but not unpaired trained rats. **(C)** Conditioned taste aversion memory extinction is significantly slower in animals that were trained previously for fear conditioning compared to animals trained with unpaired protocol or left naïve. The analysis revealed a significant main effect for groups (χ^2^_(2)_ = 10.162; *p* < 0.007). There is interaction between groups and time variables (χ^2^_(2)_ = 12.712; *p* < 0.003). Analysis between groups revealed that there is an interaction between the fear conditioning and naïve groups (χ^2^_(1)_ = 7.583, *p* < 0.007), between the fear conditioning and unpaired groups (χ^2^_(1)_ = 6.295, *p* < 0.02) but not between the unpaired and naïve groups (χ^2^_(1)_ = 0.013, *p* > 0.9). **(D)** Conditioned taste aversion memory retrieval is not affected by fear conditioning. Animals were trained for fear conditioning or left naïve. Four days later animals were trained for CTA using lower concentration of LiCl (3 ml of 0.015 M LiCl) to study whether fear conditioning can enhance CTA memory retrieval. Conditioned taste aversion memory was not significantly different between fear-conditioned or naïve trained animals (*p* > 0.3) showing that CTA memory retrieval is not affected by fear conditioning.

Fear conditioning took place in a Plexiglas rodent conditioning chamber with a metal grid floor. Rats were habituated to the training chamber (context A) for 3 days. Animals were presented with five pairings of a tone for 40 s as the CS (5 kHz, 80 dB) that was co-terminated with a foot shock as the US (0.5 s, 1.3 mA). The intertrial interval (ITI) was random with average of 180 s. Unpaired training took place in the same conditioning chamber. Rats received non-overlapping five presentations of the CS and US where the US preceded the CS by 60 s and at least 120 s was required between a tone CS and the next trial. The naïve group was introduced to the training cage with no CS or US. Rat groups were tested 24 h after training for long-term memory in a different chamber with different context and Formica floor (context B), to diminish the effect of context. Animals were presented with two tones (40 s, 5 kHz, 80 dB) with average ITI of 180 s. Behavior was recorded and the video images were transferred to a computer equipped with an analysis program. The percentage of changed pixels between two adjacent 0.5 s images was used as a measure of activity. The experimental data shown in Figures 1, 3 includes only rats whose freezing was above 50% following cued fear conditioning and below 35% after unpaired training in the second test tone.

**Figure 2:**
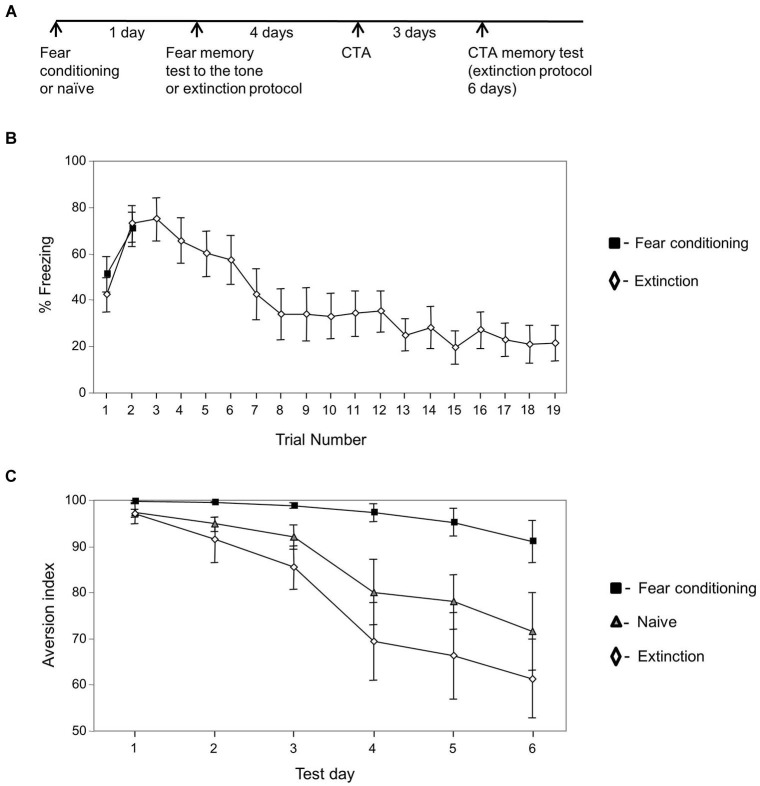
**Extinguished fear conditioning memory has no effect on CTA memory extinction**. **(A)** Experimental timeline: animals were trained for fear conditioning or left naïve (*n* = 10). The next day the fear conditioned trained animals were divided into two groups: the first was tested for fear memory to the tone (two tones (*n* = 8)) and the second subjected to the fear conditioning extinction protocol (*n* = 12). Four days later the animals were subjected to CTA training. Three days later they were tested for taste memory once each day for 6 days. **(B)** Freezing during fear memory test and during fear memory extinction protocol. Freezing was not different between groups on the second tone (*p* > 0.5) but was significantly and markedly reduced in the extinction group on the 19th tone when compared to the second tone between groups (*p* < 0.003) or within the extinction group (*p* < 0.001). **(C)** Conditioned taste aversion memory extinction is significantly slower in animals that were trained previously for fear conditioning compared with animals trained with fear memory extinction protocol or left naïve. The analysis revealed significant main effect for group (χ^2^_(2)_ = 14.886; *p* < 0.002). There is an interaction between groups and time variables ((χ^2^_(2)_ = 19.915; *p* < 0.001). Analysis between groups revealed that there is an interaction between fear conditioning and naïve groups (χ^2^_(1)_ = 5.630, *p* < 0.02), between fear conditioning and extinction groups (χ^2^_(1)_ = 16.054, *p* < 0.001) but not between extinction and naïve groups (χ^2^_(1)_ = 1.256, *p* > 0.2).

**Figure 3:**
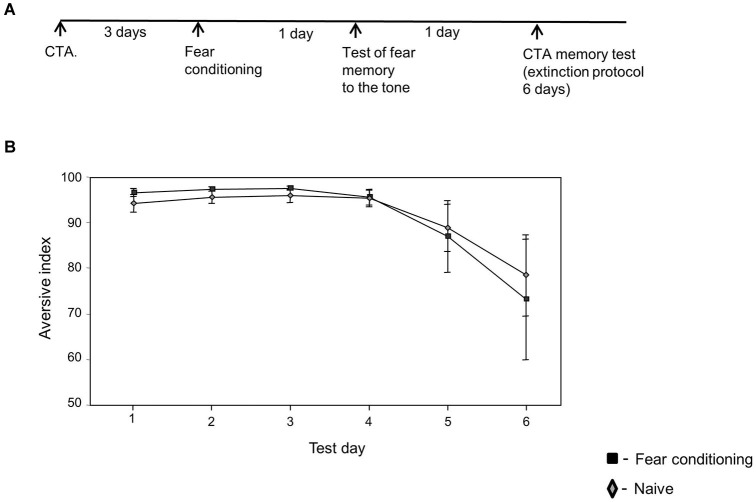
**Fear conditioning has no effect on CTA memory extinction if CTA memory was formed before fear conditioning learning. (A)** Experimental time line. Rats were trained for CTA memory. Three days later they were divided into two groups that were trained with fear conditioning (*n* = 5) or left without training (naïve, *n* = 8). The next day the animals were tested for fear memory formation. Twenty four hours later the animals were tested for CTA memory extinction. **(B)** No difference in CTA memory extinction was detected between the naïve and fear conditioning trained rats (χ^2^_(1)_ = 0.02, *p* > 0.9).

### Fear conditioning extinction

**Figure 4:**
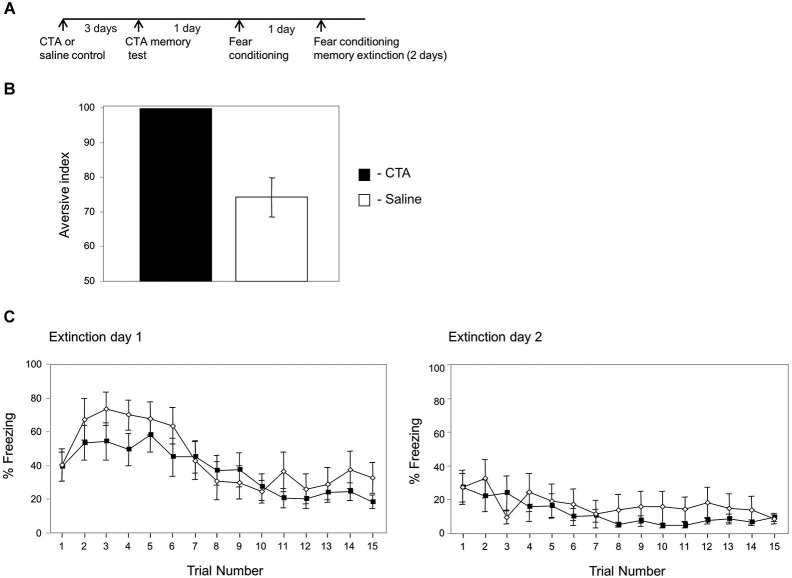
**Conditioned taste aversion training has no effect on fear memory extinction**. **(A)** Experimental timeline: animals were paired with saccharin and LiCl (CTA; *n* = 11) or saccharin and saline (control; *n* = 10). Three days later they were tested for taste aversion memory. The next day they were trained for fear conditioning. The following next 2 days the animals were subjected to the fear memory extinction protocol. **(B)** Taste aversion is significantly higher in the CTA group compared to the control (*p* < 0.001) showing that the CTA trained animals formed taste aversion memory. **(C)** Fear memory extinction is not different between animals subjected to CTA or controls in both test days. In the first test day there is no main effect for group (CTA, saline controls) (χ^2^_(1)_ = 2.037, *p* > 0.15) and no interaction between group and time variables (χ^2^_(1)_ = 0.062, *p* > 0.8). In the second test day there is no main effect for group (CTA, saline controls) (χ^2^_(1)_ = 0.091, *p* > 0.7) and no interaction between group and time variables (χ^2^_(1)_ = 0.101, *p* > 0.7).

Fear conditioning was performed as above. The next day animals were subjected to presentations of 19 CSs (Figure 2) or 15 CSs (Figure 4) (40 s, 5 kHz, 80 dB, ITI 180) in a different chamber with different context and Formica floor (context B), to diminish the effect of context.

### Conditioned taste aversion

Rats were trained over 3 days to get their daily water ration within 20 min/day from two pipettes, each containing 10 ml. On day 4 (conditioning day), the rats were presented with saccharin (0.1% w/v, sodium salt; CS) instead of water. Sixty minutes later, they were injected with LiCl (3 ml 0.07 M LiCl; US) intraperitoneally. For CTA memory retrieval (Figure 1D) animals were injected with lower concentration of LiCl (3 ml 0.015 M LiCl). On days 5–6, the rats were presented daily for 20 min with two pipettes containing 10 ml of water each. Conditioned taste aversion memory test was performed on six successive days (days 7–12) where the rats were presented daily with an array of four pipettes, two containing 10 ml of saccharin and two containing 10 ml water for 20 min. Their liquid consumption was recorded and aversion index was calculated. The aversion index was defined as [milliliters of water/(milliliters of water + milliliters of saccharin)] × 100 consumed in the test; that is, 50 is chance level, and the higher the aversive index the strongest memory is where rats remember the CS as an aversive stimulus.

### Statistics

Repeated-measures analysis using the generalized estimating equations (GEE) approach was performed (Zeger and Liang, 1986). Generalized estimating equations was used instead of ANOVA since, in some cases, the assumptions of equality of covariance matrix and of multivariate normality of residuals of our data were not met. The GEE approach is especially robust to misspecification of variance/covariance structure. Furthermore, GEE assumes that the correlations among measures across time are not of direct interest, and focuses on the comparison of groups across time. Single comparison between two groups was done using Mann-Whitney U test. Statistical analysis was done using the SPSS 20 software.

## Results

### Cued fear conditioning impairs the extinction of future memory

We were interested to explore the possibility that fearful experience leading to long-term fear memory could affect the extinction of a different memory. Toward that end we trained rats for fear conditioning and studied its effects on subsequent CTA memory extinction (Figure 1A). Animals were trained for fear conditioning to associate a tone (CS) with a footshock (US). Control animals received the same sensory stimulation but in an unpaired non-associative manner or were naïve and not subjected to the CS and US. The paired protocol (fear conditioning) consistently leads to auditory fear conditioning memory formation, whereas the unpaired protocol does not. Long-term conditioned fear memory was assessed by measuring freezing responses elicited by the CS without the US 24 h after conditioning. Figure 1B shows that the paired rats froze significantly more than the unpaired controls (χ^2^_(1)_ = 87.779; *p* < 0.003). Four days later animals were trained for CTA and were tested 3 days afterwards for long-term taste memory for six consecutive days (Figure 1C). Results that compared the effect of group (fear conditioning, unpaired and naïve groups) and time (CTA test days) were analyzed. The analysis revealed a significant main effect for group (χ^2^_(2)_ = 10.162; *p* < 0.007). There is interaction between groups and time variables (χ^2^_(2)_ = 12.712; *p* < 0.003). Analysis between groups revealed that there is an interaction between the fear conditioning and naïve groups (χ^2^_(1)_ = 7.583, *p* < 0.007), between the fear conditioning and unpaired groups (χ^2^_(1)_ = 6.295, *p* < 0.02) but not between the unpaired and naïve groups (χ^2^_(1)_ = 0.013, *p* > 0.9) showing that CTA memory in the previously fear conditioning trained animals extinguished significantly slower than in control groups. In addition, the results show that this phenomenon is not affected by acute stress induced by the unpaired training.

### Fear conditioning has no effect on CTA memory retrieval

In aforementioned results we observed that fear conditioning impaired CTA memory extinction. This leaves the possibility that fear conditioning may enhance the level of the subsequent taste aversion memory rather than impairing extinction. To test this possibility we trained rats for fear conditioning followed by a weak CTA protocol (same protocol as in Figure 1A but with weaker CTA). Using weaker CTA protocol results in weaker CTA memory (see Figure 1D) and provides the possibility for fear learning to enhance CTA memory. We show that fear conditioning had no effect on CTA memory retrieval (*p* > 0.3, Figure 1D). Fear conditioning has therefore no effect on CTA memory retrieval but specifically on its extinction.

### Extinguished fear memory has no effect on CTA memory extinction

We were interested to further explore whether training for fear conditioning has an irreversible effect on taste memory extinction. If fear learning has an irreversible effect on taste memory extinction then fear memory extinction after fear conditioning training should have no effect on the impairments exerted by fear conditioning learning on taste memory extinction (as observed in Figure 1C). Two new groups of animals were trained for fear conditioning or left naïve. The next day the fear conditioned trained animals were divided into two groups: the first was tested for fear memory to the tone (two tones) and the second subjected to the fear conditioning extinction protocol (19 tones) (Figure 2A). Figure 2B shows the freezing responses of fear conditioning only group and of animals that underwent fear memory extinction protocol 1 day after fear conditioning training. Freezing was markedly reduced in the extinction group on the 19th tone when compared to the second tone (*p* < 0.001). Four days later the animals were trained for CTA and subjected 3 days afterwards to CTA memory tests for six consecutive days (Figure 2C). Results that compared the effect of group (fear conditioning, extinction of fear conditioning, and naïve groups) and time (CTA memory test days) were analyzed. The analysis revealed significant main effect for group (χ^2^_(2)_ = 14.886; *p* < 0.002). There is an interaction between groups and time variables (χ^2^_(2)_ = 19.915; *p* < 0.001). Analysis between groups reveals that there is an interaction between fear conditioning and naïve groups (χ^2^_(1)_ = 5.630, *p* < 0.02), between fear conditioning and extinction groups (χ^2^_(1)_ = 16.054, *p* < 0.001) but not between extinction and naïve groups (χ^2^_(1)_ = 1.256, *p* > 0.2) showing that CTA memory extinction in previously fear conditioning trained animals is significantly slower than in animals that their fear memory was extinguished before CTA training and naïve animals. It also shows that CTA memory of the fear extinction group extinguishes similarly to CTA memory of the naïve rats.

### Fear conditioning has no effect on CTA memory extinction if CTA memory was formed before fear learning

We were interested to study whether fear conditioning could affect the extinction of memories that were formed before fear learning. Rats were trained for CTA 3 days before fear conditioning and were tested for CTA memory extinction 2 days after fear conditioning training. Figure 3 shows that fear conditioning has no effect on CTA memory extinction (χ^2^_(1)_ = 0.02, *p* > 0.9) if CTA memory was formed before fear learning. This shows that fear conditioning affects specifically the extinction of memories formed after, but not before, fear learning.

### CTA memory has no effect on fear conditioning memory extinction

Next we were interested to understand whether the effects of fear conditioning on CTA memory extinction is a typical effect of one memory on the other. Toward that end, we studied the effects of CTA on fear memory extinction (timeline in Figure 4A). Rats were divided to two groups the first trained for CTA and the second control group was trained to drink saccharin but received saline instead of LiCl. The animals were tested once 3 days later for CTA memory. As shown in Figure 4B the CTA trained animals are significantly more aversive to saccharin than control animals injected with saline (*p* < 0.001). The next day the animals were subjected to fear conditioning. The next 2 days the animals were tested for fear conditioning memory extinction (Figure 4C). There is no main effect for group (CTA, saline controls) (χ^2^_(1)_ = 2.037, *p* > 0.15) and no interaction between group and time variables (χ^2^_(1)_ = 0.062, *p* > 0.8) in the first fear extinction test day. Similarly, there is no main effect for group (CTA, saline controls) (χ^2^_(1)_ = 0.091; *p* > 0.7) and no interaction between group and time variables (χ^2^_(1)_ = 0.101; *p* > 0.7) in the second day of fear memory extinction. These results show that CTA has no effect on fear memory extinction and indicate that the effects of fear conditioning on memory extinction are not general effects between two types of memories.

## Discussion

In this study we show that cued fear conditioning impairs specifically the extinction, but not the retrieval, of different memory formed for CTA. We further show that this effect is specific to CS-US association and is not affected by the US or CS when presented unpaired. Furthermore, the results show that extinction of fear memory eliminates the effects of fear conditioning on CTA memory extinction. Fear conditioning affects CTA memory extinction only if CTA memory was formed after and not before fear conditioning learning. The creation of CTA memory has no effect on fear conditioning memory extinction.

Our study shows that cued fear conditioning training of a tone paired with the footshock affects CTA memory extinction. Unpaired training where the footshock and tone do not overlap and are presented in a non-associative manner had no effect on CTA memory extinction. These results show that the CS and US *per se* have no effect on memory extinction. Footshock *per se* produces stressful responses such as strong activation of the hypothalamic-pituitary-adrenal (HPA) axis (Dagyte et al., 2009). Stress can affect CTA memory extinction when subjected during the extinction trials (Akirav et al., 2009). In addition, stressors can affect CTA memory when subjected 30 min before (Bourne et al., 1992), during (Bourne et al., 1992; Misanin et al., 2006) or 15 min after (Bourne et al., 1992) CTA training or as the US (Dess et al., 1998; Brand et al., 2008). We did not observe any effect on CTA extinction when the footshock was presented unpaired days before CTA but only as the US in fear conditioning. This observation indicates that the stressful responses produced by the footshock have no effect on CTA memory extinction and that cued fear memory formation specifically affects the extinction of CTA memory.

Fear conditioning leads to long-term fear memory to the tone whereas unpaired training do not. It is possible that the fear conditioned tone CS affects neurons that are involved in the extinction of CTA memory. Memory extinction of fear conditioning in animals leads to increased responses of neurons in prefrontal cortex compared to same extinction protocol in pseudorandom (unpaired) trained group (Barrett et al., 2003). Conditioned taste aversion memory extinction requires the prefrontal cortex (e.g., Akirav et al., 2006) and CTA memory extinction leads to an increase in neuronal responses to the taste CS in prefrontal cortex (Mickley et al., 2005). It is therefore possible that fear conditioning, but not unpaired training, leads to alteration in prefrontal cortex neurons that can affect future CTA memory extinction. It is also possible that the neurons involved in CTA memory extinction in the prefrontal cortex receive regulatory information during CTA memory extinction from amygdala neurons modified by fear conditioning. The BLA sends direct excitatory projections to the IL (McDonald, 1991; Pérez-Jaranay and Vives, 1991; Condé et al., 1995). It was shown that the BLA controls the learned auditory fear-induced changes in neuronal activity in the mPFC (Garcia et al., 1999). The BLA also modulates responses in the prefrontal cortex during CTA memory extinction (e.g., Xin et al., 2014). The BLA is needed for CTA memory extinction. For example, it was shown that when the protein translation inhibitor anisomycin or the β-adrenergic receptor antagonist propranolol are microinjected into the BLA on the first day of CTA memory extinction they impair extinction when compared to saline injected controls (Bahar et al., 2003). In addition, microinjection of the GABAA receptor agonist, muscimol, into the BLA immediately after the first CTA extinction session disrupts the extinction of CTA (Akirav, 2007). Activity of cells in BLA is altered during CTA memory extinction. For example, c-Fos protein level, which serves as a marker for neuronal activity, in BLA changes during CTA memory extinction (Mickley et al., 2004). Studies detected neurons in the BLA that are multimodal responding to both taste and auditory stimuli (e.g., Nishijo et al., 1998). Thus, it could be that auditory responsive neurons altered by fear conditioning in LA can affect extinction of CTA memory through regulating prefrontal cortex neurons. Indeed, fear conditioning leads to increased response in LA neurons whereas unpaired training shows little or no change (e.g., Repa et al., 2001). Thus, increased neuronal response in LA after fear conditioning, but not after unpaired training, could potentially affect CTA extinction by controlling prefrontal cortex neurons. This can explain the significant differences we observed between the fear conditioning and unpaired groups in affecting CTA memory extinction.

The rate of extinction is sensitive to the strength of the original learning. This leaves open the possibility that fear conditioning enhanced the level of the subsequent taste aversion rather than impairing its extinction. We therefore tested the effect of fear conditioning on the strength of the original learning. Toward that end we trained the animals with a weaker CTA protocol that leads to weaker CTA memory and allows to test whether fear conditioning can enhance CTA memory. We found that fear conditioning has no effect on CTA memory strength as there is no differences on CTA memory retrieval. We conclude that fear conditioning affects specifically CTA memory extinction but not CTA memory formation.

Next we asked whether the specific alteration created by fear conditioning that can affect different memory extinction is irreversible after fear conditioning training or could it be adjusted. Fear memory to the tone is attenuated by exposure to the tone without the shock during the extinction procedure (Milad and Quirk, 2012). If neuronal alteration after fear conditioning training, leading to changes in CTA memory extinction, is permanent then extinction of fear memory after fear conditioning training will have no effect on impairments of CTA memory extinction. We revealed that fear memory extinction abolished the effects of fear conditioning training on CTA extinction. This observation indicates that the alterations of neuronal circuits by fear conditioning that can affect CTA extinction are adjustable.

Evidence shows that extinction does not erase the initial association between the CS and US but rather forms a new association (CS-No US) that inhibits expression of the conditioned memory. Thus, fear memory for the CS still exists after extinction but is inhibited. Evidence supporting the fact that the fear memory for the CS still exist after extinction shows that the original inhibited fear memory can reemerge in rodents and humans after: (1) renewal, when the CS is presented outside of the extinction context (Robbins, 1990; Effting and Kindt, 2007); (2) reinstatement, when the original US is given unexpectedly (Rescorla and Heth, 1975; Bouton and Bolles, 1979; Westbrook et al., 2002; Schiller et al., 2008); or (3) spontaneous recovery, when a substantial amount of time has passed (Robbins, 1990; Schiller et al., 2008). In our study we show that fear extinction abolished the effects on CTA memory extinction. Thus, although memory for the fearful event exists after fear memory extinction its inhibition eliminates its ability to affect CTA memory extinction. Therefore, our finding shows that fear memory needs to be active and not inhibited to affect CTA memory extinction.

We also observed that training for CTA has no effect on fear memory extinction. This observation show that the brain system that mediates CTA memory formation do not leave a trace, as does fear conditioning training, that can affect fear memory extinction.

In this study we show that a specific fearful experience leads to alteration of extinction of a different memory. Such an adjustment in the rate of extinction may be useful for better behavioral response and evaluation of information in possibly dangerous environment. Slower extinction rate provides the organism with an additional time to examine and evaluate the novel information to ensure its safety.

## Author contributions

Gil Joels designed, interpreted and executed all experiments; Raphael Lamprecht designed and interpreted all the experiment and wrote the manuscript.

## Conflict of interest statement

The authors declare that the research was conducted in the absence of any commercial or financial relationships that could be construed as a potential conflict of interest.
